# A locked immunometabolic switch underlies TREM2 R47H loss of function in human iPSC‐derived microglia

**DOI:** 10.1096/fj.201902447R

**Published:** 2019-12-23

**Authors:** Thomas M. Piers, Katharina Cosker, Anna Mallach, Gabriel Thomas Johnson, Rita Guerreiro, John Hardy, Jennifer M. Pocock

**Affiliations:** ^1^ Department of Neuroinflammation University College London Queen Square Institute of Neurology London UK; ^2^ Center for Neurodegenerative Science Van Andel Research Institute Grand Rapids MI USA; ^3^ Department of Neurodegenerative Diseases University College London Queen Square Institute of Neurology London UK

**Keywords:** Alzheimer's disease, glycolysis, metabolism, microglia

## Abstract

Loss‐of‐function genetic variants of *triggering receptor expressed on myeloid cells 2 (TREM2)* are linked with an enhanced risk of developing dementias. Microglia, the resident immune cell of the brain, express TREM2, and microglial responses are implicated in dementia pathways. In a normal surveillance state, microglia use oxidative phosphorylation for their energy supply, but rely on the ability to undergo a metabolic switch to glycolysis to allow them to perform rapid plastic responses. We investigated the role of TREM2 on the microglial metabolic function in human patient iPSC‐derived microglia expressing loss of function variants in TREM2. We show that these TREM2 variant iPSC‐microglia, including the Alzheimer's disease R47H risk variant, exhibit significant metabolic deficits including a reduced mitochondrial respiratory capacity and an inability to perform a glycolytic immunometabolic switch. We determined that dysregulated PPARγ/p38MAPK signaling underlies the observed phenotypic deficits in TREM2 variants and that activation of these pathways can ameliorate the metabolic deficit in these cells and consequently rescue critical microglial cellular function such as β‐Amyloid phagocytosis. These findings have ramifications for microglial focussed‐treatments in AD.

**Abbreviations**
2‐DG: 2‐deoxyglucose
ECAR: extracellular acidification rate
iPSC: induced pluripotent stem cell
iPS‐Mg: induced pluripotent stem cell‐derived microglia
LOAD: late‐onset Alzheimer’s disease
Nasu Hakola Disease: NHD
OCR: oxygen consumption rate
PFKFB3: 6‐phosphofructo‐2‐kinase/fructose‐2,6‐biphosphatase 3
PGC‐1α: peroxisome proliferator‐activated receptor gamma coactivator 1‐alpha
PPARγ: peroxisome proliferator‐activated receptor gamma
TREM2: triggering receptor expressed on myeloid cells 2

## 1 INTRODUCTION

Microglia are the tissue‐resident macrophages and innate immune cells of the brain and need to rapidly respond to changes in their environment. Quiescent, surveillant innate immune cells such as microglia present in the normal brain respond to activating stimuli by reprograming metabolism, specifically, switching to favor glycolysis over oxidative phosphorylation.^1^ Whilst glycolysis is an inefficient way to generate ATP, this switch enables microglia to undergo the rapid changes associated with their enhanced plasticity of function.^2^ It is also the preferential mechanism in proliferative cells to facilitate the uptake and incorporation of essential nutrients vital for the production of new cellular biomass.^3^

In neurodegenerative diseases, aberrant microglial function has been increasingly recognized to contribute to disease progression; however, the influence of the cellular metabolic phenotype is not well understood. Missense mutations of *triggering receptor expressed on myeloid cells 2 (TREM2)* are associated with an enhanced risk of developing dementia including late‐onset Alzheimer’s disease (LOAD).^4^, ^5^ In the CNS, TREM2 is exclusively expressed in microglia and numerous studies have linked the disease‐associated mutations to deficits in microglial function, including ligand binding/sensing, phagocytosis, and inflammatory responses.^6^, ^7^

Much of the current work to elucidate the loss of functional consequences of TREM2 variants in AD has employed the use of KO animal models and whilst a role for TREM2 has been described in microglial metabolism,^8^ it is not known whether disease‐relevant variants also harbor metabolic deficits or the nature of any observed deficits. Here, we used human iPSC‐derived microglia (iPS‐Mg) generated from donors harboring specific TREM2 mutations previously characterized as hypomorphic variants in Alzheimer’s disease and Nasu Hakola disease (NHD), and identified deficits in microglial metabolic regulation and associated functions. Furthermore, we identified for the first time that TREM2 variants are unable to carry out an immunometabolic switch to induce glycolysis and that this depends on PPARγ‐p38MAPK‐PFKFB3 signaling.

## 2 MATERIALS AND METHODS

### 2.1 iPSC generation

Ethical permission for this study was obtained from the National Hospital for Neurology and Neurosurgery and the Institute of Neurology joint research ethics committee (study reference 09/H0716/64) or by the Ethics Committee of Istanbul Faculty of Medicine, Istanbul University (for the collection of T66M mutant fibroblasts to Dr Ebba Lohmann). R47H heterozygous fibroblasts were acquired with a material transfer agreement between University College London and University of California Irvine Alzheimer’s Disease Research Center (UCI ADRC; M Blurton‐Jones). Fibroblast reprograming was performed by episomal plasmid nucleofection (Lonza) as previously described,^9^ using plasmids obtained from Addgene (#27077, #27078 and #27080). Nucleofected cultures were transferred to Essential 8 medium (Life Technologies) after 7 days in vitro (DIV) and individual colonies were picked after 25‐30 DIV and CNV analysis was performed (Supplementary Figure 1A). All iPSCs were maintained and routinely passaged in Essential 8 medium. Karyotype analysis was performed by The Doctors Laboratory (London, UK) (Supplementary Figure 2B‐D). The R47H^hom^ line was a gene‐edited isogenic of BIONi010‐C, purchased from EBiSC (BIONi010‐C7). Control iPSC lines used in this study are as follows: CTRL1 (kindly provided by Dr Selina Wray, UCL Institute of Neurology); CTRL2 (SBAD03, StemBANCC); CTRL3 (SFC840, StemBANCC); CTRL4 (BIONi010‐C, EBiSC).

### 2.2 iPSC‐derived microglia (iPS‐Mg)

Using our previously described protocol, iPSC‐microglia (iPS‐Mg) were generated.^10^ Experimental replicates were either individual donors (control lines and R47H^het^ lines), or separate clones of the same donor (T66M^het^, T66M^hom^, and W50C^hom^ lines), or one clone assayed in independent experimental runs (R47H^hom^), due to the rarity of patient or genome‐edited samples.

### 2.3 Microglia gene array

A custom gene array based on published microglial expression data^11^, ^12^, ^13^, ^14^, ^15^ (Table 1) was used to confirm a microglial signature in our iPS‐Mg (TaqMan™ Array Plate 32 plus Candidate Endogenous Control Genes; Thermo Fisher Scientific). Complementary DNA was generated from iPS‐Mg, iPSC‐derived microglial like cells,^16^ and human monocyte‐derived macrophage (hMacs) RNA samples using the High‐Capacity RNA‐cDNA kit (Life Technologies), according to the manufacturer’s instructions. Human primary microglia cDNA was also analyzed as a control sample (ScienCell). Quantitative PCR was conducted on an Mx3000p qPCR system with MxPro qPCR software (Agilent Technologies) using TaqMan™ Gene Expression Master Mix (Thermo Fisher). Heat maps were generated with the gplots^17^ and d3heatmap^18^ packages in R.

**Table 1:** TaqMan assay details used in the custom microglial gene signature array

Gene symbol	Gene name	Assay ID
*18s rRNA*	18s ribosomal RNA	Hs99999901_s1
*GAPDH*	Glyceraldehyde‐3‐phosphate dehydrogenase	Hs99999905_m1
*HPRT*	Hypoxanthine Phosphoribosyltransferase	Hs99999909_m1
*GUSB*	Glucuronidase Beta	Hs99999908_m1
*APOE*	apolipoprotein E	Hs00171168_m1
*C1QA*	complement C1q A chain	Hs00706358_s1
*C1QB*	complement C1q B chain	Hs00608019_m1
*ITGAM*	integrin subunit alpha M	Hs00167304_m1
*CSF1R*	colony stimulating factor 1 receptor	Hs00911250_m1
*CX3CR1*	C‐X3‐C motif chemokine receptor 1	Hs01922583_s1
*GAS6*	growth arrest‐specific 6	Hs01090305_m1
*GPR34*	G protein‐coupled receptor 34	Hs00271105_s1
*AIF1*	allograft inflammatory factor 1	Hs00610419_g1
*MERTK*	MER proto‐oncogene, tyrosine kinase	Hs01031979_m1
*OLFML3*	Olfactomedin‐like 3	Hs01113293_g1
*PROS1*	protein S (alpha)	Hs00165590_m1
*SALL1*	spalt‐like transcription factor 1	Hs01548765_m1
*SLCO2B1*	solute carrier organic anion transporter family member 2B1	Hs01030343_m1
*TGFBR1*	transforming growth factor beta receptor 1	Hs00610320_m1
*TMEM119*	transmembrane protein 119	Hs01938722_u1
*TREM2*	triggering receptor expressed on myeloid cells 2	Hs00219132_m1
*BIN1*	bridging integrator 1	Hs00184913_m1
*CD33*	CD33 molecule	Hs01076282_g1
*SPI1*	Spi‐1 proto‐oncogene	Hs02786711_m1
*HEXB*	hexosaminidase subunit beta	Hs01077594_m1
*ITM2B*	integral membrane protein 2B	Hs00222753_m1
*C3*	complement component 3	Hs00163811_m1
*A2M*	alpha‐2‐macroglobulin	Hs00929971_m1
*C1QC*	complement C1q C chain	Hs00757779_m1
*RGS1*	regulator of G‐protein signaling 1	Hs01023772_m1
*FTL*	ferritin light chain	Hs00830226_gH
*P2RY12*	purinergic receptor P2Y12	Hs01881698_s1

### 2.4 Cellular stress proteome array

Cells were treated for 8 hours with 2‐deoxyglucose (2‐DG; 3mM) and cell lysates were prepared as per the manufacturer’s instructions (Proteome Profiler™ Human cell stress array; Bio‐Techne). Total protein quantification was performed on the aliquots of each treatment group for data normalization purposes. Lysates were pooled from three independent experiments, according to the *TREM2* genotype after basal or 2‐DG treatment. Data were analyzed using the Protein Array Analyser Palette plugin for ImageJ,^19^ and plotted as relative protein expression, normalized to total cellular protein levels.

### 2.5 Immunoblotting

iPS‐Mg were lysed in RIPA buffer (50 mM Tris, 150mM NaCl, 1% SDS, and 1% Triton X‐100) containing 1× Halt™ protease and phosphatase inhibitor cocktail. Lysates were separated into soluble and insoluble (nuclear) fractions. Samples were resolved and transferred onto https://www.sciencedirect.com/topics/biochemistry-genetics-and-molecular-biology/nitrocellulose membranes and incubated with primary and secondary antibodies (Table 2). Blotting was visualized on an Odyssey detection system (LI‐COR) and quantified using ImageJ software (http://www.imagej.nih.gov/ij).

**Table 2:** Primary and secondary antibody details

Target	Clone	Cat No	Supplier	Dilution
Actin	AC‐15	A5441	Sigma	1:10 000
PPARγ	C26H12	2435	Cell Signaling Labs	1:1000
TREM2	D8I4C	91 068	Cell Signaling Labs	1:1000
pS571‐PGC1α		AF6650	Novus Biologicals	1:500
PGC1α		NBP1‐04676	Novus Biologicals	1:1000
pT180/Y182‐P38 MAPK	D3F9	4511P	Cell Signaling Labs	1:2000
P38 MAPK		9212	Cell Signaling Labs	1:1000
pT334‐MAPKAPK2	27B7	3007	Cell Signaling Labs	1:1000
MAPKAPK2		3042	Cell Signaling Labs	1:1000
PFKFB3		ab181861	Abcam	1:1000
Goat anti‐rabbit IgG H&L (IRDye® 800CW)		ab216773	Abcam	1:10 000
Goat anti‐mouse IgG (H + L) Alexa Fluor 680		A21058	Thermo Fisher	1:10 000

### 2.6 Live cell morphological staining

iPS‐Mg were matured on 13 mm glass coverslips. Cells were treated with 100 ng/mL of LPS and 10U/mL of human IFNγ for 24 hours prior to staining. Prior to visualization, cells were incubated with 1 µM Calcein‐AM for 15 minutes at 37°C. Images were captured on a Zeiss Axioskop 2 fluorescence microscope and image analysis was carried out with AxioVision 4.8 and ImageJ software.

### 2.7 Mitochondrial superoxide analysis

Basal levels of mitochondrial superoxide were measured by loading cells with MitoSOX**™** red superoxide indicator (Thermo Fisher) and analyzed by flow cytometry. Briefly, a 5µM working concentration of MitoSOX**™** red was prepared in warmed FACs buffer (PBS + 0.5% BSA). Cells (400 000) were incubated with MitoSOX**™** red for 10 minutes at 37°C, protected from light. Cells were washed twice and resuspended in FACs buffer followed by flow cytometry (FL2; FACs Calibur, Beckton Dickinson). Incubation with Rotenone (100 nM) for 30 minutes was used as a positive control. Data were analyzed using Flowing Software v2.5.1 (University of Turku).

### 2.8 Mitochondrial number determination

To assess gross mitochondrial number in the iPS‐Mg lines, flow cytometry analysis using MitoTracker**™** Green (Thermo Fisher) was performed. Briefly, a 200 nM working concentration of MitoTracker**™** Green was prepared in warmed FACs buffer (PBS + 0.5% BSA). Cells (400 000) were incubated with MitoTracker**™** Green for 30 minutes at 37°C, protected from light. Cells were washed twice and resuspended in FACs buffer followed by flow cytometry (FL1; FACs Calibur, Beckton Dickinson). Data were analyzed using Flowing Software v2.5.1 (University of Turku).

### 2.9 PPARγ transcriptional activity

The DNA binding activity of PPARγ was detected in iPS‐Mg nuclear extracts as previously described^20^ from untreated and groups treated with 2‐DG (3mM) for 8 hours, using a PPARγ transcription factor assay kit, as per the manufacturer’s instructions (Abcam).

### 2.10 Cellular respiration analysis

For the real‐time analysis of oxygen consumption rates (OCR) and extracellular acidification rates (ECAR), iPS‐Mg were plated and matured on Seahorse cell culture microplates and analyzed using a Seahorse XFe96 Analyser (Agilent Technologies). Cells were incubated overnight with or without pioglitazone (100 nM) or GW0742 (100 nM). Mito stress kits were used to analyze mitochondrial respiration and Glycolytic stress kits were used to analyze cellular glycolysis. Data were analyzed using Wave v2.4.0.6 software (Agilent Technologies).

### 2.11 6‐phosphofructokinase (PFK) activity assay

The activity of the glycolysis enzyme PFK was assessed in iPS‐Mg cellular lysates after treatment with pioglitazone (100nM) for 24 hours ± pretreatment with SB202190 (100 nM; 1 hour prior), as per the manufacturer's instructions (Abcam).

### 2.12 Aβ_1‐42_ (HiLyte488) phagocytosis

Cells were plated and matured at a density of 20 000/well in 24 well plates (2 wells pooled/treatment group). Cells were treated with pioglitazone (100 nM) ± pretreatment (1 hour prior) with SB202190 (100 nM) or AZ‐PFKFB3 (50nM) for 24 hours prior to FACs analysis. On the day of the experiment, cells were removed from the incubator to equilibrate to RT for 30 minutes and cytochalasin‐D (CytoD; 100 µM) was added to negative control groups. Aβ_1‐42_ (HiLyte488; 100 nM) was added to all groups except unstained groups and allowed to bind to phagocytic receptors for 30 minutes at RT. Grouped wells were then pooled into 2ml tubes, centrifuged at 300 *g* for 3 minutes at RT, media aspirated, and the cell pellet resuspended in fresh RT maturation medium. Tubes were incubated at 37°C + 5% CO_2_ for 1 hour to initiate phagocytosis of bound Aβ_1‐42_. Tubes were centrifuged at 300 *g* for 3 minutes at RT, media was aspirated, and cell pellets were resuspended in PBS for FACs analysis (FL1; FACs Calibur, Beckton Dickinson). Data were analyzed using FCS Express 6 Plus (De Novo Software).

## 3 RESULTS

### 3.1 TREM2 variant human iPS‐Mg exhibit reduced oxidative phosphorylation and glycolytic capability

**Figure 1:**
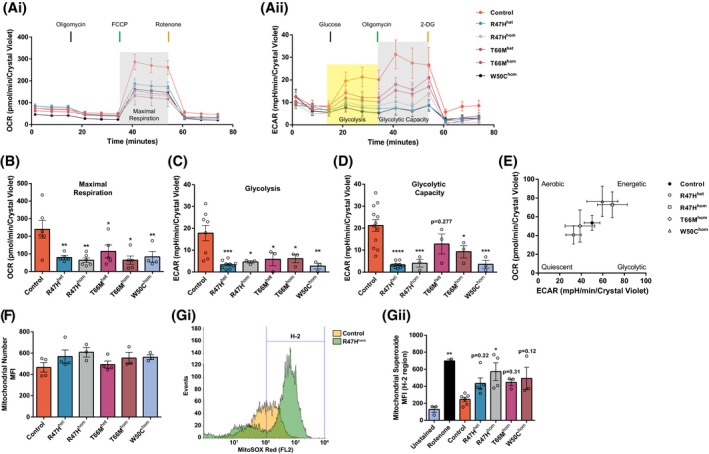
Significant deficits in cellular respiration are observed in iPS‐Mg from TREM2 hypomorphic lines. The oxygen consumption rate (OCR) (Ai) and the extracellular acidification rate (ECAR) (Aii) were analyzed in control iPS‐Mg and TREM2 mutant iPS‐Mg to assay mitochondrial oxidative function and the glycolytic function of the lines, respectively. Analysis of iPS‐Mg maximal respiration, post FCCP injection, identified significant deficits in the OCR of TREM2 hypomorphic lines, when compared with controls (B); analysis of iPS‐Mg glycolysis, post glucose injection, identified significant deficits in the ECAR of TREM2 hypomorphic lines, when compared with controls (C); analysis of iPS‐Mg glycolytic capacity, post oligomycin injection, identified significant deficits in the ECAR of TREM2 hypomorphic lines, when compared with controls (D). The basal metabolic phenotype of iPSC parental lines used to develop iPS‐Mg is independent of TREM2 genotype (E). MitoTracker Green flow cytometry of control and TREM2 hypomorphic iPS‐Mg suggested that the observed deficits in cellular respiration were not due to differences in mitochondrial number. Plotted as mean fluorescent intensity (MFI; F). Representative histogram of MitoSOX™ red flow cytometry in control and R47H^hom^ iPS‐Mg (Gi) and quantification of basal mitochondrial reactive oxygen species (mean fluorescence intensity, MFI ± SEM) (Gii) show significantly higher levels in TREM2 hypomorphic mutants when compared to control levels. Data are represented as mean ± SEM (n *≥ *3). Statistical significance was addressed using 1‐way ANOVA with Bonferroni’s multiple comparison test to compare control iPS‐Mg with TREM2 hypomorphic iPS‐Mg lines or positive control, *P < .05; **P < .01; ***P < .005; ****P < .001

Using our previously described techniques^10^ we generated human iPS‐Mg harboring polymorphisms implicated in Alzheimer’s disease (AD; R47H) and in Nasu Hakola disease (NHD; T66M/W50C) via mesodermal germ layer induction and primitive hematopoiesis (Supplementary Figure 2A‐C), with comparable microglial genetic profiles in multiple lines (Supplementary Figure 2D) harboring polymorphisms that give rise to differential TREM2 protein glycosylation and cleavage (Supplementary Figure 2E). Cellular respiration by Seahorse analysis of the oxygen consumption rate (*OCR*) and extracellular acidification rate (*ECAR*) was investigated in all available lines. Under mitochondrial stress and glycolytic stress, deficits were observed in oxidative phosphorylation (Figure 1Ai) and glycolytic function (Figure 1Aii), respectively. Specifically, levels of maximal respiration (Figure 1B), glycolysis (Figure 1C), and glycolytic capacity (Figure 1D) were significantly reduced in all iPS‐Mg harboring TREM2 variants compared with control lines. We confirmed that respiratory deficits were not due to underlying differences in the parental iPSC lines (Figure 1E); the results indicate that all lines prior to differentiation (and before TREM2 expression) displayed metabolic phenotypes independent of TREM2 genotype. We also measured mitochondrial number, since it has been reported that TREM^‐/‐^ mice exhibit reduced mitochondrial mass^8^; however, we found no difference as measured by MitoTracker Green between control and TREM2 variant iPS‐Mg lines (Figure 1F). Instead, the production of mitochondrial superoxide assessed by MitoSOX red was modestly increased in both AD R47H and NHD TREM2 iPS‐Mg (Figure 1Gi,Gii), suggesting the possible dysregulation of basal mitochondrial function in TREM2 mutant carriers.

### 3.2 iPS‐Mg with TREM2 hypomorphic variants fail to “switch” to glycolysis following an inflammatory challenge

**Figure 2:**
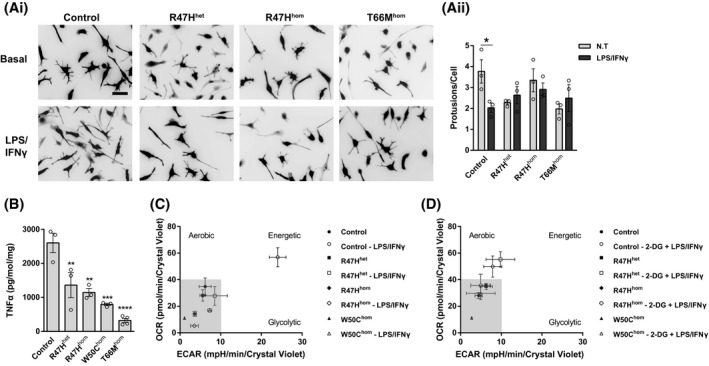
Activated morphologies and release of TNFα were reduced in TREM2 hypomorphic iPS‐Mg. Representative images (Ai) and quantification of cellular protrusions (Aii) on iPS‐Mg after LPS/IFNγ treatment shows a reduction of activated morphologies in TREM2 mutant lines. ELISA analysis of LPS/IFNγ‐treated iPS‐Mg identified a significant reduction in TNFα release from TREM2 mutant lines (B). Metabolic phenotypes of iPS‐Mg after LPS/IFNγ treatment show a loss of a metabolic switch in TREM2 mutant lines (C). Metabolic phenotypes of iPS‐Mg after the inhibition of glycolysis and LPS/IFNγ treatment show a loss of switch to active oxidative phosphorylation in TREM2 homozygous mutant lines (D). In (Ai), scale bar: 50 μm. Data are presented as mean ± SEM (n ≥ 3). Statistical significance was addressed using 1‐way ANOVA with Bonferroni’s multiple comparison test to compare control iPS‐Mg with TREM2 hypomorphic iPS‐Mg lines, **P < .01; ***P < .005; ****P < .001, or 2‐way ANOVA with Bonferroni’s multiple comparison test to compare nontreated and LPS/IFNγ groups, *P < .05

Given the energetic deficits in TREM2 variant iPS‐Mg in response to mitochondrial and glycolytic stress, we investigated how these cells respond to an inflammatory challenge. We found that following exposure to lipopolysaccharide (LPS) and interferon‐gamma (IFNγ), control iPS‐Mg undergo morphological changes, specifically increased cellular elongation and a reduction in ramified processes (Figure 2Ai,Aii), and show a substantial release of the pro‐inflammatory cytokine TNFα (Figure 2B). In TREM2 variant iPS‐Mg, the morphological changes are less dramatic (Figure 2Ai,Aii) and they exhibit a significant reduction in TNFα release compared with control lines (Figure 2B), indicating a deficit in the microglial response to the inflammatory stimulus. Microglia respond to pro‐inflammatory stimuli by initiating a metabolic switch from oxidative phosphorylation to glycolysis. Seahorse analysis was again employed to measure *OCR* and *ECAR,* this time following exposure to LPS and IFNγ. Control iPS‐Mg showed a robust shift from low quiescent to high energetic respiration in response to LPS and IFNγ (Figure 2C). In contrast, TREM2 variant iPS‐Mg from AD and NHD lines remained “stuck” in a quiescent respiratory state (Figure 2C). When glycolysis was blocked with 2‐deoxyglucose (2‐DG, 3mM) prior to LPS/IFNγ, control iPS‐Mg are still able to generate aerobic energy (Figure 2D) to compensate. Interestingly, R47H^het^ iPS‐Mg were also able to induce aerobic respiration following the inhibition of glycolysis, unlike the other TREM2 homozygous variants that remained in a quiescent state (Figure 2D).

### 3.3 TREM2 variant iPS‐Mg induce cellular stress pathways in response to glycolytic inhibition and exhibit reduced PPARγ signaling

**Figure 3:**
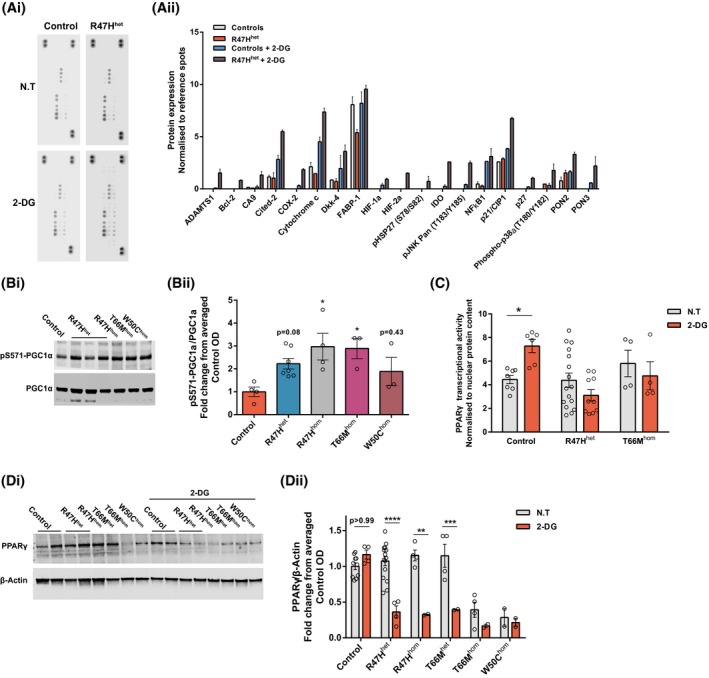
iPS‐Mg with TREM2 hypomorphs have enhanced cellular stress indicators and aberrant PPARγ signalling. Representative cellular stress proteome array dot blots from control and R47H^het^ iPS‐Mg cell lysates +/− 2‐DG (3mM) treatment (Ai) and quantification of cell stress proteome array show increased levels of several cellular and mitochondrial‐associated stress proteins in R47H^het^ iPS‐Mg lysates after 2‐DG treatment (Aii). Representative western blotting of phospho‐S571‐PGC1α and total PGC1α protein levels in control and mutant TREM2 iPS‐Mg (Bi) and quantification of the protein levels identified enhanced the levels of phospho‐S571‐PGC1α in TREM2 mutant expressing iPS‐Mg lines (Bii). Cellular stress induced by 2‐DG (3mM) enhanced PPARγ transcriptional activity in control iPS‐Mg but not in R47H^het^ or T66M^hom^ iPS‐Mg; control: *P* = .0372, R47H^het^: *P* = .3352, T66M^hom^: *P* > .9999; n = 4‐16, normalized to nuclear protein content ± SEM (C). Representative western blotting of PPARγ and β‐Actin protein levels in control and mutant TREM2 iPS‐Mg after 2‐DG treatment (Di) and quantification identified reductions in PPARγ protein levels in TREM2 mutant iPS‐Mg after 2‐DG treatment, or in the case of T66Mhom and W50Chom, reduced basal levels of the protein compared to control levels (Dii). Data are presented as mean ± SEM (n ≥ 3). Statistical significance was addressed using 1‐way ANOVA with Bonferroni’s multiple comparison test to compare control iPS‐Mg with TREM2 hypomorphic iPS‐Mg lines (Bii) or 2‐way ANOVA with Bonferroni’s multiple comparison test to compare nontreated and 2‐DG groups (C, Dii), *P < .05; **P < .01; ***P < .005; ****P < .001

In order to investigate the comparable aerobic responses after glycolytic inhibition, but underlying phenotypic differences between the control and patient AD R47H^het^ iPS‐Mg, cells were treated with 2‐DG and probed for changes in key cell stress pathways. In 2‐DG‐treated R47H^het^ iPS‐Mg, we identified enhanced levels of cell stress proteins that are linked to energy metabolism and/or mitochondrial function, including HIF1α, cytochrome‐c, Bcl‐2, Cited‐2, NF‐κB, and p38α MAPK (Figure 3Ai,Aii). PGC‐1α, the master regulator of mitochondrial biogenesis and energy metabolism,^21^, ^22^ is the downstream of a number of these pathways and so we examined phosphorylation levels at serine 571, which negatively regulates PGC‐1α activity in control and TREM2 variant iPS‐Mg. We found that phosphorylation at serine 571 was significantly enhanced in TREM2 variant iPS‐Mg, suggesting that PGC‐1α activity is downregulated in TREM2 variant cells (Figure 3Bi,Bii). PGC‐1α is also a coactivator with the nuclear receptor and transcription factor PPARγ widely expressed in inflammatory cells such as microglia and macrophages as well as adipose tissue where it controls inflammation, lipid metabolism, and glucose homeostasis.^23^ Following 2‐DG treatment, we found increased PPARγ transcriptional activity in control iPS‐Mg but no increase in AD R47H^het^ or NHD T66M^hom^ iPS‐Mg (Figure 3C) despite the increase in cell stress pathways in AD R47H^het^ iPS‐Mg. When we looked at total PPARγ protein levels in iPS‐Mg, we found that PPARγ protein levels were significantly reduced in TREM2 hypomorphs, either at basal (T66M^hom^/W50C^hom^) or after glycolytic inhibition with 2‐DG (R47H^het/hom^ and T66M^het^) (Figure 3Di,Dii).

### 3.4 A PPARγ agonist can rescue energy deficits and the metabolic glycolytic switch in TREM2 variant iPS‐Mg

**Figure 4:**
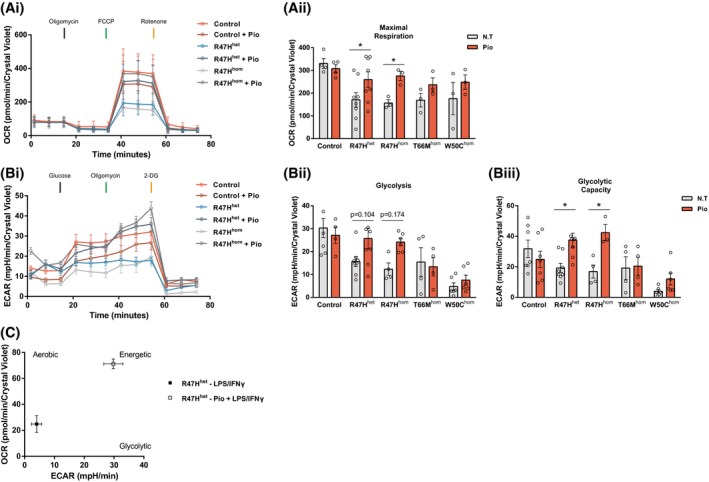
Activation of PPARγ signaling rescues cellular respiration and metabolic phenotype switch in AD‐associated R47H iPS‐Mg lines. Complete traces of the control and R47H hypomorphic iPS‐Mg mitochondrial stress test pretreated with 100 nM pioglitazone (Ai). Pretreatment with pioglitazone reversed the maximal respiration deficits observed in R47H iPS‐Mg lines when compared with nontreated groups (Aii). Complete traces of the control and R47H hypomorphic iPS‐Mg glycolytic stress test pretreated with 100 nM pioglitazone (Bi). Pretreatment with pioglitazone partially reversed the glycolysis deficits (Bii) and significantly reversed glycolytic capacity deficits (Biii) in R47H lines. The LPS/IFNγ‐induced metabolic switch is rescued in R47H^het^ iPS‐Mg after the activation of PPARγ (C). Data are presented as mean ± SEM (n ≥ 3). Statistical significance was addressed using 2‐way ANOVA with Bonferroni’s multiple comparison test to compare nontreated with treated groups within genotypes, *P < .05

We set out to determine whether dysregulation of PPARγ signaling was the cause of the inability of TREM2 hypomorphs to switch to an energetic phenotype and whether this could be rescued by targeting PPARγ activation. We thus investigated whether oxidative phosphorylation and glycolysis could be modulated through PPARγ agonism; preincubation with the PPARγ agonist pioglitazone significantly attenuated the observed energy deficits in maximal respiration **(**Figure 4Ai,Aii), glycolysis (Figure 4Bi,Bii), and glycolytic capacity (Figure 4Bi Biii) in iPS‐Mg from R47H^het/hom^ carriers, but interestingly did not significantly enhance maximal metabolic respiration in control iPS‐Mg, suggesting that metabolic function in these cells was already optimal or saturated. In the case of the T66M^hom^, or W50C^hom^ TREM2 hypomorphic iPS‐Mg, whilst there was a trend to increased respiration with pioglitazone, this was not significant (Figure 4Aii) and pioglitazone did not enhance glycolysis in these cells either (Figure 4Bii,Iii). Since the R47H variant expressing iPS‐Mg were most amenable to rescue with pioglitazone treatment, we examined whether this activation could promote the switch to an energetic phenotype following inflammatory stimulation with LPS and IFNγ; indeed, this was found to be the case (Figure 4C), suggesting that we can reverse the metabolic deficit in these cells.

### 3.5 Pioglitazone rescues the energy deficits in TREM2 variant iPS‐Mg through p38‐MAPK and PFKFB3 signaling

**Figure 5:**
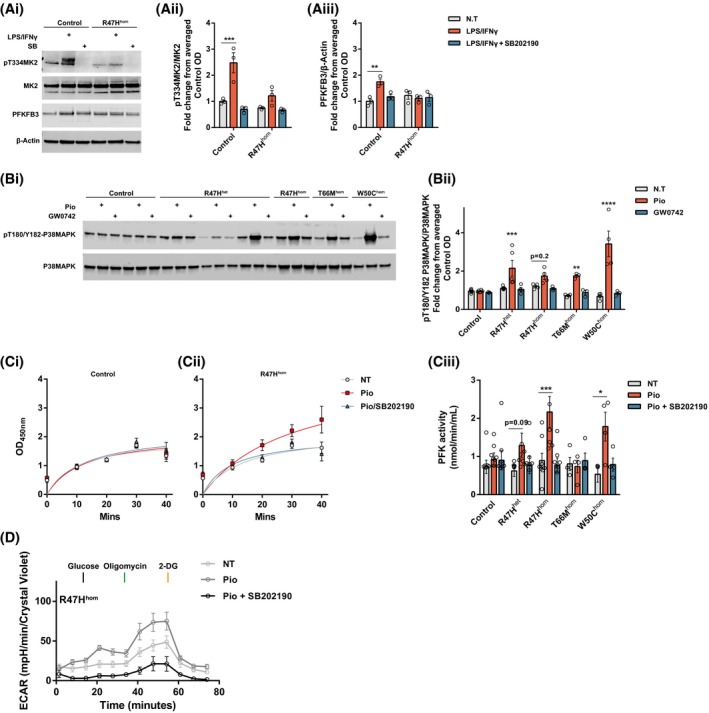
Pioglitazone rescues the energy deficits in TREM2 variant iPS‐Mg through p38‐MAPK and PFKFB3 signaling. Representative western blotting of phospho‐T334‐MK2, total MK2, and PFKFB3 protein levels in control and mutant TREM2 iPS‐Mg after LPS/IFNγ ± SB202190 (Ai) and quantification identified enhanced levels of phospho‐T334‐MK2 (Aii) and PFKFB3 protein (Aiii) after LPS/IFNγ treatment specifically in control lines, when compared to R47Hhom iPS‐Mg, and was dependent on P38MAPK signaling. Representative western blotting of phospho‐T180/Y182‐P38MAPK and total P38MAPK protein levels in control and mutant TREM2 iPS‐Mg after pioglitazone or GW0742 treatment (Bi) and quantification identified the enhanced levels of phospho‐T180/Y182‐P38MAPK after pioglitazone treatment specifically in TREM2 hypomorphic iPS‐Mg lines (Bii). PFK1 enzyme activity is specifically enhanced by pioglitazone in TREM2 hypomorphic lines when compared to control iPS‐Mg, and is dependent on p38MAPK (Ci‐Ciii). Complete traces of the R47H^hom^ iPS‐Mg glycolytic stress test show that pioglitazone is able to enhance glycolysis and glycolytic function in a P38MAPK‐dependent manner (D). Data are presented as mean ± SEM (n ≥ 3). Statistical significance was addressed using 2‐way ANOVA with Bonferroni’s multiple comparison test to compare LPS/IFNγ, or pioglitazone treatment of each TREM2 variant with the corresponding nontreated group, *P < .05; **P < .01; ***P < .005; ****P < .001

To further investigate the mechanisms by which pioglitazone can initiate the switch to glycolysis in TREM2 R47H variants, we probed the involvement of the PPARγ target PFKFB3 (Guo et al., 2010),^24^ a key regulatory enzyme in glycolysis.^25^, ^26^ PFKFB3 regulates glucose metabolism via the synthesis of fructose‐2,6‐bisphosphate, a potent allosteric activator of 6‐phosphofructo‐1‐kinase (PFK‐1), which catalyzes the committed step of glycolysis through the conversion of fructose‐6‐phosphate and ATP to fructose‐1,6‐biphosphate and ADP.^27^ The signaling pathway leading to the activation of PFKFB3 is through p38‐MAPK‐dependent phosphorylation of MK2 and subsequent phosphorylation of PFKFB3 for the induction of the glycolytic switch.^28^, ^29^ We again used LPS/IFNγ to induce glycolysis in iPS‐Mg and found increased phosphorylation of MK2 and total protein levels of PFK3B3 that is dependent on p38‐MAPK activity (Figure 5Ai‐Aiii). However, in R47H^hom^ iPS‐Mg there is a substantial deficit in the ability of LPS/IFNγ to induce phospho‐MK2, again suggesting that these cells cannot induce glycolysis after an inflammatory challenge (Figure 5Ai‐Aiii). Given that p38‐MAPK was increased in R47H in response to glycolytic stress (Figure 3A), it was surprising that p38‐MAPK‐dependent phosphorylation of MK2 was decreased in response to LPS/IFNγ. We, therefore, investigated the effect of PPARγ activation by pioglitazone on basal p38MAPK in unstimulated iPS‐Mg. We found a strong induction of pT180/Y182‐p38MAPK by western blot after pioglitazone exposure (100nM) in TREM2 variant iPS‐Mg compared with control lines, suggesting that PPARγ induces a significant activation of the p38‐MAPK pathway to rescue glycolysis in TREM2 lines that is not observed in control lines (Figure 5Bi,Bii). Activation of PPARδ/β by GW0742 was ineffective at increasing p38MAPK phosphorylation (Figure 5Bii), suggesting this pathway is specific to PPARγ. We also probed the PFK‐1 activity downstream of PFKFB3 and shown that pioglitazone is able to enhance PFK‐1 activity, but only in the TREM2 hypomorphic lines and that this is p38‐MAPK dependent (Figure 5Ci‐Ciii), again supporting the idea that TREM2 variants can be rescued via the activation of a p38‐MAPK pathway to induce glycolysis. Indeed, when we probed glycolysis by Seahorse in R47H^hom^ iPS‐Mg, inhibition of p38MAPK blocked the rescue of cellular glycolytic function by pioglitazone activation of PPARγ (Figure 5D).

### 3.6 Activation of the metabolic glycolytic switch by pioglitazone rescues the deficit in phagocytosis of Aβ_1‐42_ in TREM2 variant iPS‐Mg

**Figure 6:**
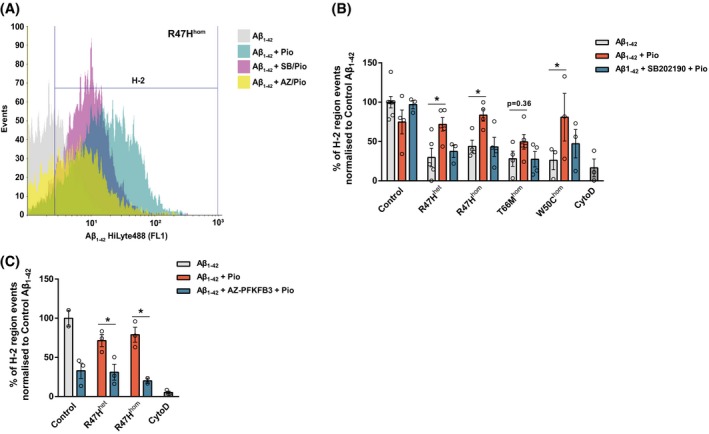
PPARγ‐mediated rescue of microglial functional deficits is dependent on p38MAPK signaling. Representative histogram of R47H^hom^ iPS‐Mg‐mediated phagocytosis of Aβ_1−42_ HiLyte488 after treatment with pioglitazone ± P38MAPK or PFKFB3 inhibition (A). Quantification of Aβ_1−42_ phagocytosis in iPS‐Mg identified that pioglitazone treatment enhanced phagocytosis in TREM2 variant lines and inhibition of P38MAPK prevented the observed rescue (B). Quantification of Aβ_1−42_ phagocytosis in control and R47H variant iPS‐Mg identified that inhibition of PFKFB3 prevented the pioglitazone‐mediated rescue of phagocytosis in the TREM2 variant lines (C). Data are presented as mean ± SEM (n ≥ 2). Statistical significance was addressed using 2‐way ANOVA with Bonferroni’s multiple comparison test to compare Aβ_1−42_ + pioglitazone treatment of each TREM2 variant to the corresponding Aβ_1−42_ group (B) or to compare Aβ_1−42_ + pioglitazone treatment to Aβ_1−42_ + AZ‐PFKFB3 inhibition + pioglitazone treatment in each corresponding group (C), *P < .05

Since pioglitazone can rescue the metabolic switch to glycolysis in TREM2 variant iPS‐Mg, we asked whether PPARγ‐p38MAPK/PFKFB3 activation can also rescue TREM2‐dependent cellular functions. TREM2 has been shown to be important for phagocytosis, a crucial function of microglia that is found to be aberrant in neurodegenerative diseases such as Alzheimer’s.^6^, ^30^ We have shown previously that NHD‐associated TREM2 variant iPS‐Mg have a deficit in phagocytosis of apoptotic cells^31^; however, this is not observed in R47H (data not shown). We, therefore, investigated the ability of TREM2 variants to clear amyloid beta (Aβ), which accumulates in plaques in AD and is the major pathological hallmark of the disease. Here we show that the R47H and NHD variants exhibit a substantial deficit in their ability to phagocytose oligomeric Aβ_1‐42_ compared with control cells (Figure 6A‐C). Since phagocytosis has been shown to be driven in macrophages by glycolytic metabolism,^32^ we determined whether pioglitazone could reverse the reduced phagocytosis of Aβ in TREM2 variant iPS‐Mg. Activation of PPARγ by pioglitazone significantly rescues the TREM2 variant deficit in R47H lines and this increase in phagocytosis is dependent on p38MAPK signaling (Figure 6A,B) and PFKFB3 activity (Figure 6A,C). These data suggest that the recovery of the glycolytic switch by the activation of PPARγ‐p38MAPK/PFKFB3 signaling is sufficient to rescue TREM2‐dependent microglial functions such as phagocytosis of Aβ in AD.

## 4 DISCUSSION

Recent findings in mouse models suggest that TREM2 plays a role in maintaining microglial metabolic fitness^8^ and that TREM2 modulates the metabolic homeostasis of adipose tissue‐associated macrophages specifically the homeostasis of glucose, insulin, cholesterol, HDL, and LDL^33^ as well as disease‐associated microglia in AD.^34^

Our data point to significant deficits in human iPS‐Mg harboring TREM2 hypomorphic variants when in a basal homeostatic state. Specifically, these microglial lines exhibit reduced maximal mitochondrial respiratory capacity as well as reduced glycolytic capacity when compared with common variant controls. We confirm that these deficits correspond to the presence of TREM2 variants and are not an inherent property of the iPSC since the corresponding undifferentiated cells do not show TREM2 genotype‐dependent metabolic deficits. These energy deficits are not due to lower mitochondrial numbers, but may be influenced by enhanced mitochondrial superoxide levels. Indeed, mitochondrial impairment, namely energy deficiencies and aberrant ROS signaling, has been previously linked to several neurodegenerative diseases,^35^ most notably Parkinson’s disease^36^ but also AD and aging.^37^, ^38^, ^39^ Our current findings are the first to show these metabolic deficits in disease relevant and human TREM2 hypomorphic microglia. However, whilst microglia upon activation can produce a burst of reactive oxygen species via the activity of the NAPDH oxidase^40^, ^41^ it remains to be seen whether the production of cellular superoxide is altered in TREM2 hypomorphic microglia.

We identified aberrant responses following exposure to classical pro‐inflammatory stimuli (LPS/IFNγ) in TREM2 hypomorphic iPS‐Mg; specifically, reduced morphological changes and TNFα release. Both responses require rapid energy production which is produced when microglia undergo the metabolic switch to glycolysis.^26^, ^32^ Glycolysis allows energy production and uptake of essential nutrients to support the rapid changes required by “activated” microglia in response to a stimulus, such as phagocytosis, proliferation, migration, and induction of protein synthesis for cytokine and chemokine secretion.^3^, ^42^, ^43^, ^44^ Indeed, we identified the ability of the TREM2 variant expressing iPS‐Mg to undergo a normal switch in metabolism from a homeostatic, surveillance profile supported by oxidative phosphorylation, to one in which glycolysis is impaired. Conversely, the control iPS‐Mg responded to the pro‐inflammatory stimuli in a similar manner to recent studies that have employed primary microglia cultures.^26^, ^45^ Interestingly, when we blocked glycolysis prior to LPS/IFNγ exposure, only control and R47H^het^ iPS‐Mg were able to increase energy demand through enhanced oxidative phosphorylation. These data suggest a less dramatic loss of energy production in the AD‐associated risk variant compared with the more severe NHD mutations.

The identification of comparable aerobic responses after glycolytic inhibition, but underlying metabolic and phenotypic differences between the control and patient AD R47H^het^ iPS‐Mg, led us to probe the effect of glycolytic inhibition on cell stress pathways. Indeed, previous studies have shown that glycolytic inhibition can enhance oxidative stress^46^, ^47^ and with increased mitochondrial ROS levels in the TREM2 hypomorphic lines, we hypothesized that the discrepancies identified in the R47H^het^ lines may be due to a hypersensitivity to glycolytic inhibition. We found increased expression in the number of proteins indicative of metabolic and mitochondrial stress pathways in the R47H^het^ iPS‐Mg when compared with control iPS‐Mg supporting this hypothesis. Furthermore, when we investigated PGC1α, a master regulator of mitochondrial biogenesis and energy metabolism and signaling molecule downstream of a number of the pathways identified, we found enhanced negative regulation in the TREM2 hypomorphic lines. Collectively, these data suggest that whilst cell stress pathways are enhanced in R47Hhet lines during glycolytic inhibition, the cells’ ability to respond is downregulated. Indeed, when we looked at the transcriptional activity of PPARγ, a nuclear receptor and transcription factor coactivated by PCG‐1α, we found that control iPS‐Mg were able to respond to glycolytic inhibition whereas R47H^het^ and T66M^hom^ iPS‐Mg were locked and unable to respond. PPARγ is widely expressed in inflammatory cells such as microglia and macrophages as well as adipose tissue where it controls inflammation, lipid metabolism, and glucose homeostasis.^23^ In support of the aberrant PPARγ signaling, we found that protein levels were highly regulated by glycolytic inhibition, specifically in R47H lines and the heterozygous variant of T66M. Interestingly basal levels of PPARγ were significantly reduced in the severe NHD lines.

PPARγ, also known as the glitazone receptor or nuclear receptor subfamily 3 (NR1C3), is involved in insulin sensitization and enhanced glucose metabolism and plays a key role in cellular homeostasis.^48^ The prominent isoform expressed in inflammatory cells is PPARγ3. Pioglitazone, an agonist of PPARγ, has been shown to halt progression of Parkinsonism in a rodent model, ostensibly by inhibiting microglial inflammation and proliferation,^49^ although it is difficult to conclude if this is a direct effect on microglia given that the compound can affect a number of cells in these models. Targeting PPARγ for therapeutic benefit in AD has also recently been proposed.^50^ Interestingly we found that whilst the deficits in maximal respiration, glycolysis and glycolytic capacity observed in the R47H variants can be rescued by the PPARγ agonist, these deficits in the NHD hypomorphs cannot, suggesting that the reduced levels of PPARγ protein observed in these variants and the limited levels of mature TREM2 may have an uncoupling effect on the signaling to and from PPARγ. Furthermore, preincubating R47H^het^ iPS‐Mg with pioglitazone prior to LPS/IFNγ exposure rescued the energetic metabolic phenotype, strongly linking PPARγ function to the locked immunometabolic switch observed in TREM2 hypomorphic lines.

PPARγ signaling has been identified as vital in cellular immune responses. Specifically, PPARγ activation has been shown to inhibit the expression of inflammatory cytokines and promote antiinflammatory phenotypes.^51^, ^52^ Whilst PPARγ expression is reduced in NHD TREM2 hypomorphs, one might expect a correspondingly higher level of TNFα secretion if PPARγ is controlling inflammation; however, the opposite occurs. Whilst it may be true for TNFα, despite the requirement for the metabolic switch to glycolysis upon microglial activation for energy to produce cytokines and chemokines, we found previously that the ability of iPS‐Mg to produce a whole range of cytokines was not greatly impaired when a stimulus of LPS alone was used.^31^ Here we primed with IFNγ and LPS, and were able to show a deficit in the secretion of TNFα in TREM2 hypomorphs compared with controls. TNFα and IL‐6 expression may be controlled by activating transcription factor 3 (ATF3) following TLR stimulation or NFĸB and at least three classes of transcription factor and coregulators are thought to control the inflammatory secretome.^53^ Of further interest would be to determine the effects of multiple exposures to stimulants likely to evoke cytokine release in control iPS‐Mg, to determine whether there are deficiencies or enhancements in TREM2 variant iPS‐Mg.

To understand how PPARγ signaling influences the metabolic phenotype of the TREM2 variants we interrogated PFKFB3 signaling, a key regulatory enzyme involved in glycolytic induction^25^, ^26^ and identified as a target of PPARγ.^24^ We found that pioglitazone exerted its effects via a p38MAPK/PFKFB3 signaling cascade and we show that activation of the PPARγ/p38MAPK cascade and PFKFB3 activity is sufficient to rescue the functional deficit in Aβ_1‐42_ phagocytosis identified in the TREM2 hypomorphic iPS‐Mg, a key hallmark associated with AD pathogenesis. Finally, it is worth commenting on previous studies that identify p38‐MAPK activation by pioglitazone^54^, ^55^; these studies use the compound at a concentration 15‐30× higher than our treatments, which were 7× lower than the reported EC50 for PPARγ activity.

In conclusion, we find that the presence of a TREM2 mutation detrimentally influences metabolic signaling by influencing not only basal levels of oxidative phosphorylation but also the requirement of a metabolic switch to glycolysis. This inability to switch on glycolysis during a change in the environment significantly impacts the surveillant properties of homeostatic microglia leading to suboptimal responses in key microglial functions such as phagocytosis. Our data highlight these deficits in a human, genetically disease relevant microglial model and uncover a significant dependence on p38MAPK signaling during glycolysis in iPS‐Mg generated from R47H genotypes. This polymorphism seems to render the microglia hypersensitive to cellular stress; however, this susceptibility enhances the effectiveness of PPARγ activation in upregulating cellular metabolism, leading to an ability to attenuate deficits in microglial function associated with disease pathogenesis.

## CONFLICT OF INTEREST

The authors declare that they have no conflict of interest.

## AUTHOR CONTRIBUTIONS

TM Piers, JM Pocock, and J Hardy initiated the concept, TM Piers, and JM Pocock designed the experiments, and TM Piers, GT Johnson, and A Mallach carried out the experiments, R Guerreiro analyzed the in‐house generated iPSC lines, JM Pocock, TM Piers, and K Cosker wrote the paper.
