# Supplementary figures and images for: A locked immunometabolic switch underlies TREM2 R47H loss of function in human iPSC‐derived microglia
Source: FASEB J. 2019 Dec 23;34(2):2436–50. doi: 10.1096/fj.201902447R (PMC7027848; doi:10.1096/fj.201902447R)

Supp.Fig.1

A.

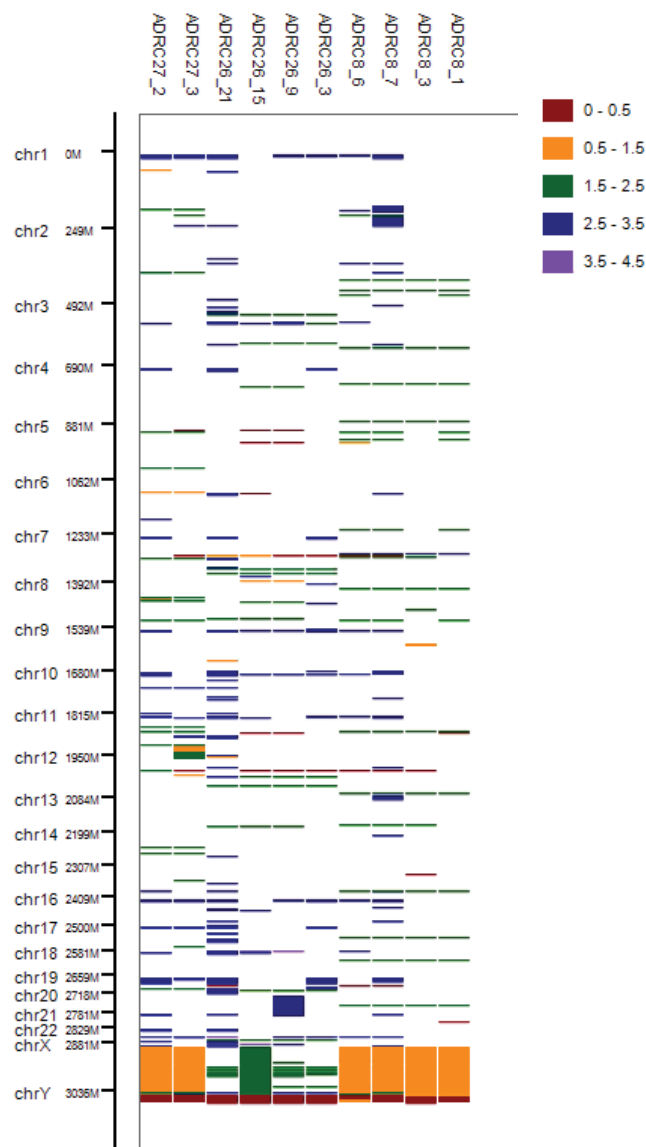

B.

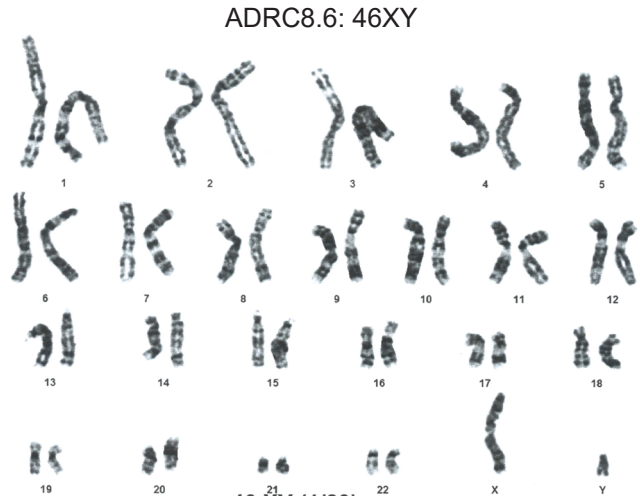

C.

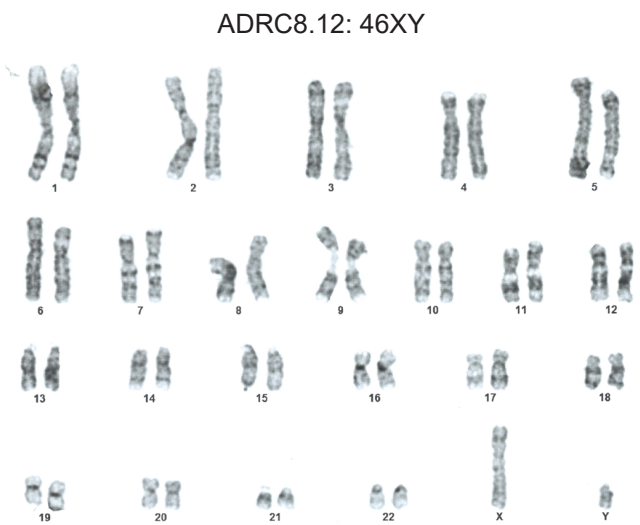

D.

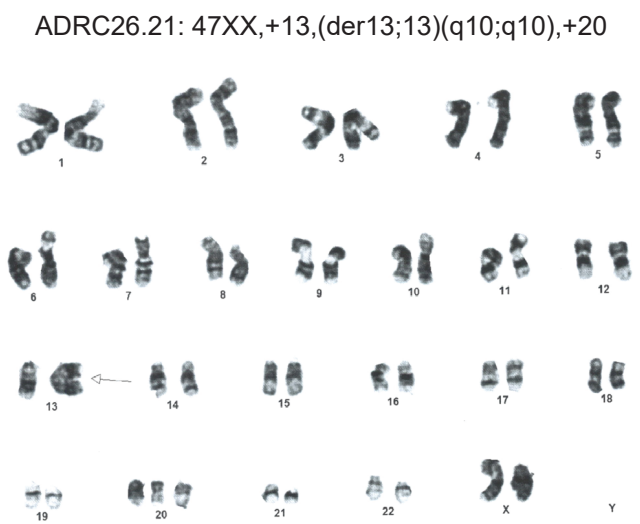

# Supp.Fig.2

**A.**

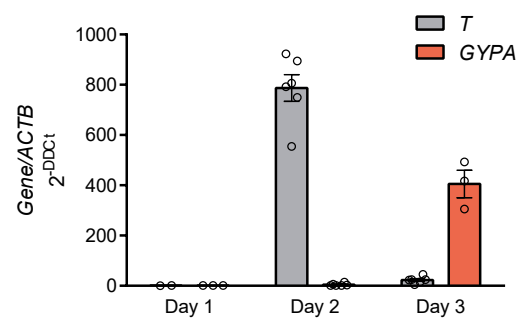

**B.**

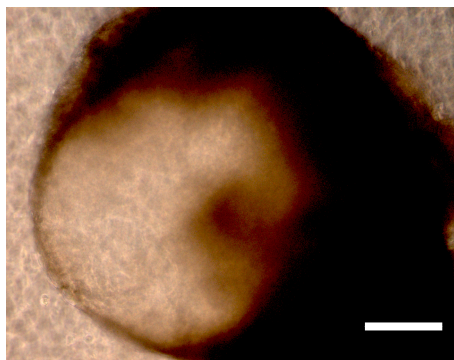

**C.**

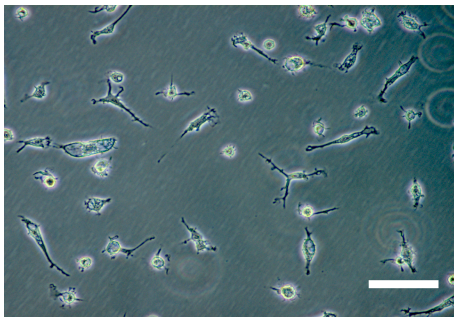

**D.**

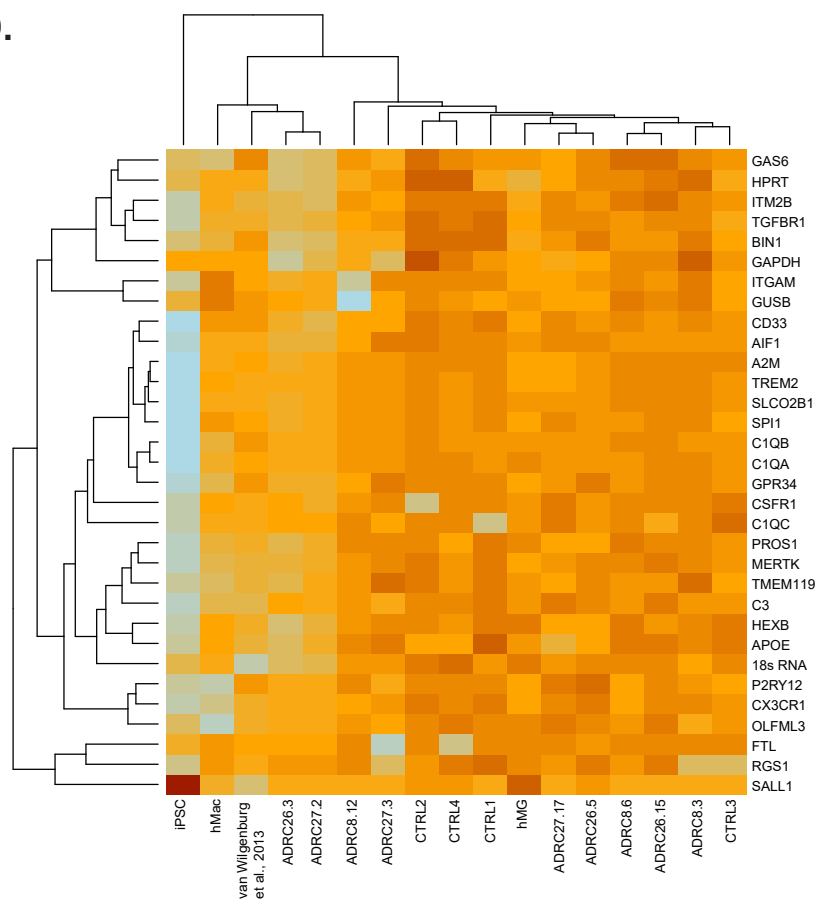

**E.**

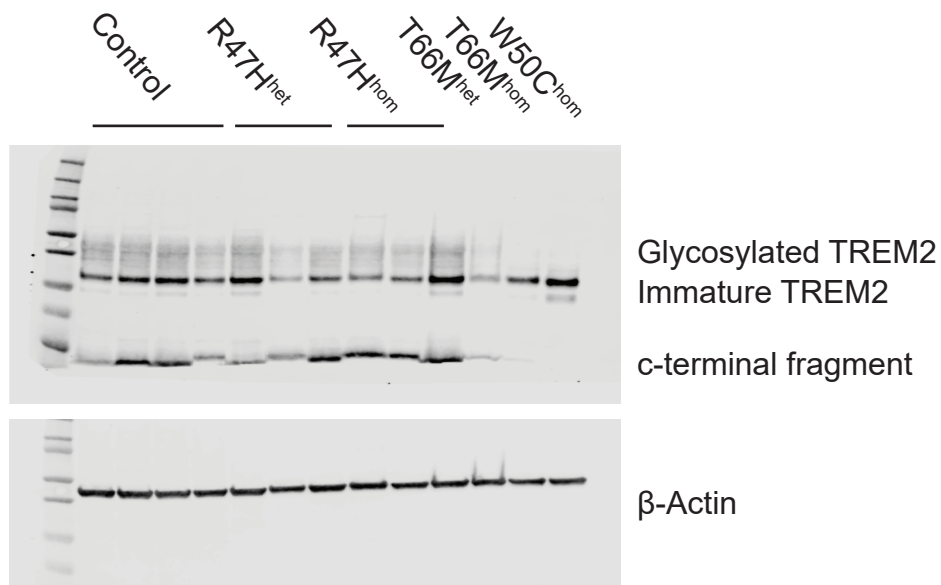

Supplement: Supplementary file 1 [file FSB2-34-2436-s001.pdf]
