# Supplementary material for: A locked immunometabolic switch underlies TREM2 R47H loss of function in human iPSC‐derived microglia
Source: FASEB J. 2019 Dec 23;34(2):2436–50. doi: 10.1096/fj.201902447R (PMC7027848; doi:10.1096/fj.201902447R)
Supplement: Supplementary file 2 [file FSB2-34-2436-s002.docx]

**Supplementary Figure Legends**

**Supp. Fig. 1. CNV and karyotype analysis of in house generated iPS-Mg variants lines. A.** Copy number variation (CNV) of iPSC clones generated from patients carrying the R47H^het^ polymorphism identified a deletion on chromosome 2 of clone 8.7, removing it from the study. Further karyotype analysis was performed on specific clones, and **B-D** provide examples.

**Supp. Fig. 2. iPS-Mg generation and characterisation. A.** Gene expression analysis of *T* and *GYPA* in embryoid bodies (EBs) after induction of primitive haematopoiesis with 3 factors (FGF2, BMP4, and VEGF) relative to *ACTB*, fold change from Day 1. **B.** Bright-field microscopy of an EB after generation in a 96 well low adherence plate. Free floating EBs form large cystic structures during culture in myeloid progenitor differentiation medium; Scale bar: 500 µm. **C.** Bright-field microscopy of fully matured iPS-Mg. Cells exhibit highly motile, dynamic, ramified processes; Scale bar: 20 µm. **D.** Heat map showing mRNA expression of a microglial gene signature in TREM2 variant iPS-Mg, iPSC-derived macrophages (van Wilgenburg et al., 2013) (<span style="baseline">15</span>) (<span style="baseline">15</span>), human monocyte-derived macrophages (hMac), human primary microglia (hMG), and iPSC samples. Clear clustering is observed between iPS-Mg and hMG, and between hMac and iPSC-derived macrophages. Two potential iPS-Mg outliers are shown to cluster with hMac and iPSC-derived macrophages. **E.** Representative western blotting of TREM2 protein levels in control and variant TREM2 lines, confirming previously published differences in TREM2 glycosylation and cleavage patterns.
